# 

**Published:** 1916-12-09

**Authors:** 


### CONTENTS.

| | PAGE |
|---|---|
| **Hospital Dietetics** ... ... ... ... ... ... | 193 |
| **The War Work of the M.O.H.** ... ... ... | 194 |
| **Hospital and Institutional News** ... ... .. | 195 |
| A New Appointment at Guy's Hospital—Tetanus in Home Military Hospitals—A Nice Point for Infirmary Managers—State Encouragement of Research—The Latest Design for an Open-air School—Extensions at King Edward VII.'s Hospital, Cardiff—In Place of the Annual Dinner—Kirkcaldy Hospital and the Nairn Family—The Irish Local Government Board and Disloyal Boards of Guardians—The Canadian Discharge Depot at Buxton—The Derby Royal Infirmary—A North of England Hospital for Incurables—Food Prices at St. Bartholomew's—Leisurely Progress in the Gowrie House Dispute—The Carnarvonshire and Anglesey Infirmary—A New Convalescent Hospital at Harrogate—This Week's Drug Market. | |
| **The Causation of Tabes and of General Paralysis** ... ... ... ... ... ... | 199 |
| A New Observation from Fiji. | |
| **A State Medical Service** ... ... ... ... | 200 |
| More Kite-Flying. | |
| **The Hospital Dietary** ... ... ... ... | 201 |
| II.—Educational Aspects. | |
| **Further Patriotism in British Hospitals** ... | 202 |
| A Group of London Medical Schools. | |
| **National Insurance Act Developments...** ... | 203 |
| Further Report of the Committee of Inquiry. | |
| **Hospital and Institutional Results in 1915** ... | 204 |
| **The Matrons' and Sisters' Department** ... | 205 |
| The Will to Destroy: Mrs. Fenwick's and the Central Committee's Statement Examined. | |
| Round the Hospitals. | |
| The Canny Scot—Mr. Stanley Justified—A Busy Christmas—Allegations to be Disregarded—Trifling with the Medical and Nursing Professions. | |
| Christmas Day: The Nurses Opportunity. | |
| **The Statement Issued by the Central Committee for State Registration in London** | 207 |
| The Committee's Attitude Defined. | |
| **Strong Protest by the Royal British Nurses' Association** ... ... ... ... ... ... ... | 208 |
| **The Editor's Letter-Box** ... ... ... ... ... | 209 |
| Trained Nurses and the Anglo-French Committee—Establishments for Massage or Special Treatment—"The Dispensary Chain in London." | |
| **Parliament and the Profession** ... ... ... | 209 |
| **The London Hospital** ... ... ... ... ... ... | 210 |
| Business of the Quarterly Court. | |
| **Matrons' and Sisters' Appointments** ... ... | vi |
| **Personalia...** ... ... ... ... ... ... | vi |
| **Passing Events and Topics** ... ... ... ... | vi |
| **The Book Market** ... ... ... ... ... ... | vi |
| **The Institutional Worker** ... ... (Suppl.) | 1 |
| Stationery Stocks in an Institution. | |
| Question for December. | |
| Enquiries and Answers. | |

